# Exogenous melatonin enhances cell wall response to salt stress in common bean (*Phaseolus vulgaris*) and the development of the associated predictive molecular markers

**DOI:** 10.3389/fpls.2022.1012186

**Published:** 2022-10-17

**Authors:** Qi Zhang, Bin Qin, Guang-da Wang, Wen-jing Zhang, Ming Li, Zhen-gong Yin, Xiankai Yuan, Hao-yue Sun, Ji-dao Du, Yan-li Du, Pengyu Jia

**Affiliations:** ^1^ College of Agriculture, Herlongjiang Bayi Agricultural University, Daqing, China; ^2^ Crop Resources Institute, Heilongjiang Academy of Agricultural Sciences, Harbin, China; ^3^ Qiqihar Branch, Heilongjiang Academy of Agricultural Sciences, Qiqihaer, China; ^4^ National Coarse Cereals Engineering Research Center, Herlongjiang Bayi Agricultural University, Daqing, China

**Keywords:** common bean, melatonin, RNA-seq, cell wall, makers, salt tolerance, regulation

## Abstract

Common bean (*Phaseolus vulgaris*) is an important food crop; however, its production is affected by salt stress. Salt stress can inhibit seed germination, promote senescence, and modify cell wall biosynthesis, assembly, and architecture. Melatonin, an indole heterocycle, has been demonstrated to greatly impact cell wall structure, composition, and regulation in plants under stress. However, the molecular basis for such assumptions is still unclear. In this study, a common bean variety, “Naihua” was treated with water (W), 70 mmol/L NaCl solution (S), and 100 μmol/L melatonin supplemented with salt solution (M+S) to determine the response of common bean to exogenous melatonin and explore regulatory mechanism of melatonin against salt stress. The results showed that exogenous melatonin treatment alleviated salt stress-induced growth inhibition of the common bean by increasing the length, surface area, volume, and diameter of common bean sprouts. Moreover, RNA sequencing (RNA-seq) and real-time quantitative PCR (qRT-PCR) indicated that the cell wall regulation pathway was involved in the salt stress tolerance of the common bean enhanced by melatonin. Screening of 120 germplasm resources revealed that melatonin treatment improved the salt tolerance of more than 65% of the common bean germplasm materials. Melatonin also up-regulated cell wall pathway genes by at least 46%. Furthermore, we analyzed the response of the common bean germplasm materials to melatonin treatment under salt stress using the key genes associated with the synthesis of the common bean cell wall as the molecular markers. The results showed that two pairs of markers were significantly associated with melatonin, and these could be used as candidate markers to predict whether common bean respond to exogenous melatonin and then enhance salt tolerance at the sprouting stage. This study shows that cell wall can respond to exogenous melatonin and enhance the salt tolerance of common bean. The makers identified in this study can be used to select common bean varieties that can respond to melatonin under stress. Overall, the study found that cell wall could response melatonin and enhance the salt tolerance and developed the makers for predicting varieties fit for melatonin under stress in common bean, which may be applied in the selection or development of common bean varieties with abiotic stress tolerance.

## Introduction

Soil salinization has become a global problem (Shabala, 2013), affecting 7% (more than 900 million hectares) of the available land worldwide. Salt stress is a limiting factor in crop production and is caused by excessive accumulation of NaCl (Wang et al., 2018). High concentrations of sodium ions (Na^+^) can reduce the osmotic potential of cells, leading to plant metabolic disorders (Fang et al., 2021). Salt stress affects plants from the seed germination stage through maturation and continues until the plant senescence. The sprouting stage has been reported to be the most sensitive to salt stress (Zhang et al., 2020). Therefore, there is an urgent need to improve the salt tolerance of plants, especially at the sprouting stage.

Plants have developed various strategies for dealing with salt stress, including the expression of salt stress-responsive genes (in ion transport, osmotic homeostasis, and toxicity resolution) and altering their physiological structures (such as lipids and cell wall) to adapt to salt stress (Zhu, 2002; Gong, 2021). The plant cell wall is a protective barrier that determines the size and shape of cells through the mechanical control of cell expansion (Carpita and Gibeaut, 1993), thus playing an integral role in salt stress tolerance (Endler et al., 2015). The cell wall is the first line of defense against salt stress (Van Zelm et al., 2020). Salt stress dehydrates plant cells, causing cellular stress (Monniaux and Hay, 2016). Overexpressing cell wall-related genes in transgenic *Arabidopsis* enhanced salt tolerance (Liu et al., 2016; Chun et al., 2019). Recent studies have shown that β-1,4-galactan (a cell wall component), whose synthesis is catalyzed by Galactan Synthase 1 (GALS1), also plays a role in salt stress tolerance. RNA-seq analysis showed that salt stress affects cell wall biosynthesis pathways (Zhang et al., 2021b). However, the relationship between cell wall biosynthesis regulation and salt stress tolerance is still unclear.

Melatonin (MT) was first discovered in the pineal gland of cattle by the physician Lerner (1959). Since its discovery, MT has been demonstrated to regulate plant growth and development processes, such as seed germination, root growth, leaf senescence, and abiotic stress responses (Arnao and Hernández-Ruiz, 2019). Moreover, MT has been shown to enhance plant stress tolerance in various ways. For example, melatonin can induce antioxidant activity, inhibit peroxidative metabolism and up-regulate ion homeostasis-related genes, such as *NHX1* and *AKT1* (Rodriguez et al., 2004; Li et al., 2012). Some studies also showed that melatonin could interact with other hormones associated with salt stress resistance in plants and stimulate the expression of defensive transcription factors (Iuchi et al., 2001; Zhao et al., 2017). Recently, special focus has been directed to the regulation of cell wall by exogenous melatonin. Melatonin content positively correlated with cell wall strength in various herbaceous peony (*Paeonia lactiflora)*, suggesting a connection between the cell wall and melatonin (Zhao et al., 2022). Exogenous melatonin could regulate cell wall biosynthesis, increase the strength of the cell wall and enhance the ability to capture ions, limiting ion levels in the cytoplasm to mitigate the toxic effects (Cao et al., 2019; Sun et al., 2021). Melatonin has been widely used in field crops such as wheat (Sun et al., 2020), soybean (Zhang et al., 2019), and cotton (Li et al., 2019); however, there are few reports about its application in the common bean.

Common bean (*Phaseolus vulgaris*), an important edible bean, is an annual legume grown in the temperate and subtropical regions. About 8,000 years ago, the common bean was cultivated only in Peru and Mexico, but it is currently grown worldwide. In 2010, the common bean planting area was about 32 million hectares, with an output of 25.42 million tons (Ganesan and Xu, 2017). However, environmental stresses, such as salt stress, have affected the growth of common bean (Popelka et al., 2004; Beaver and Osorno, 2009), necessitating its improvement for salt stress tolerance. In this study, common bean was treated with water, salt stress, and exogenous melatonin under salt stress to explore the regulatory mechanism of melatonin against salt stress. In addition, we screened 120 germplasm of common bean for salt stress tolerance. Also, the molecular markers of common bean were developed, and the valid markers associated with melatonin traits were identified. This study provides theoretical insights and application value for improving the salt tolerance of common bean at the sprouting stage through exogenous melatonin application.

## Materials and methods

### Plant materials

The common bean (*Phaseolus vulgaris*) variety ‘Naihua’ was used in this study, which was a salt-sensitive variety tested by laboratory and grown in local grown conventional variety. The validity analysis of 120 common bean germplasm varieties is listed in **Table S1**. All the germplasm materials were provided by the National Cereals Engineering Technology Research Center (NCETRE, Daqing, P.R. China).

### Plant culture conditions and treatments

The common bean seeds of similar sizes were selected and sterilized using NaClO for 5 min. The seeds were rinsed thrice with sterile distilled water. Filter papers were placed into Petri dishes and soaked with different treatment solutions to form germination beds. The seeds were then placed in the Petri dishes (20 seeds per plate) and transferred to an incubator set at 25 °C without light. Three treatments were set including distilled water (W), salt stress (S) and exogenous melatonin under salt stress (M+S). The salt stress concentration was set at 70 mmol/L (Zhang et al., 2020); The concentration of exogenous melatonin solution was 100 μmol/L. 2.3228g melatonin (WM=232.28, Coolaber, Beijing, China) was dissolved using 10mL absolute ethanol and then fixed to 1L to make the storage solution and store in the dark at 4°C. 100μL of storage solution was added in to 999.9mL 70mmol/L NaCl soultion to make the M+S treatment solution. Each treatment had five replicates. Samples were collected from the seedlings on the fifth day after the treatment. Germination indicators (such as germination rate and vitality index) were captured as described previously (Zhang et al., 2021c). The bean sprouts from the different treatments were scanned using an Epson V750 root system scanner. Morphological indicators were analyzed by WINRHIZOPRO-2004a, and images were analyzed using Image J software.

### RNA-seq analysis

The radicles of “Naihua” seedlings were selected in three biological replicates from the different treatments for RNA-Seq. Briefly, 0.5 g of the samples were used for total RNA extraction using the Versatile Plant RNA Extraction Kit (CW0581, CWBIO, Beijing, P.R. China). The RNA quality was determined by 1% agarose gel electrophoresis and NanoDrop spectrophotometer (NanoDrop™ OneC, Thermo, Massachusetts, U.S.A). RNA samples with good qualities were sent to Novogene (Beijing, P.R. China) for sequencing. Novomagic platform (https://magic.novogene.com/) was used to analyze the obtained RNA-Seq, using the “PhaVulg1_0” genome from the Ensembl database as the reference. Thereafter, DEseq (Kvam et al., 2012) was used to analyze the differentially expressed genes (DEGs) between different sample groups using the value of fold change (FC) and false discovery rate (FDR) as the screening criteria under the settings FC≥2 (log_2_FC≥1) and FDR<0.01. The enrichment of the DEGs was subsequently analyzed based on Gene Ontology (GO) and Kyoto Encyclopedia of Genes and Genomes (KEGG) databases.

### Real-time polymerase chain reaction analysis

The radicles were selected in three biological replicates from the different treatments for qRT-PCR analysis. Total RNA was extracted from the samples using Plant RNA Extraction Kit (CW0559, CWBIO, Beijing, China). The obtained RNA was used to synthesize single-strand cDNA using the *Evo M-MLV* RT Premix (AG11706, Accurate Biology, Hunan, China). The synthesized cDNA products were diluted 10-fold and then used for qRT-PCR, which was performed on a Light Cycler 480II system (Roche, Roche Diagnostics, Basel, Switzerland) using Universal qPCR SYBR Green Master Mix (11184E, Yeasen, Shanghai, China). *Pvactin11* was used as the reference gene for qRT-PCR assay (Borges et al., 2012). The qRT-PCR primers were designed by Primer Premier 5.0 software and are listed in **Table S2**. The relative expression levels of the biological and technological replicates were calculated using the delta-delta Ct (2^−ΔCt^) method (Zhang et al., 2021c).

### Construction and selection of molecular markers

The Perl script-based MISA (MIcroSAtellite) software was used to search for the genome-wide MISA sites, using the “PhaVulg1_0” genome from the Ensembl database as the reference (Peng and Lapitan, 2005). The markers were automatically designed by the Linux system-based Primer Premier software, using the design thresholds described by Qu and Liu (2013). The designed makers were then named accordingly using a Perl script. The locations of DEGs enriched in the cell wall-related GO terms and KEGG pathway were selected. The start site before and end site after 2000 bp of the DEGs were marked as the retrieval sites for screening the molecular markers.

Genomic DNA was extracted from the bean samples using the Plant Genomic DNA Extraction Kit (CW0531, CWBIO, Beijing, P.R. China). The DNA quality and quantity were determined using 1% agarose gel electrophoresis and NanoDrop spectrophotometer (NanoDropTM OneC, Thermo, Massachusetts, U.S.A). The samples were then subjected to PCR analysis using *EasyTaq* ^®^ DNA Polymerase for PAGE (AP112, Trans, Beijing, P.R. China) on a T100™ PCR machine (Hercules, California, U.S.A). Thereafter, the PCR products were separated on 9% (w/v) denaturing polyacrylamide gel (Trigiano and Caetano-Anolles, 1998).

### Statistical analysis

Analysis of variance (ANOVA) was performed at *P<0.05* significance level on the SPSS19.0 software to determine if there were significant differences between various treatments. Figures were generated using GraphPad Prism software, while the data of molecular makers were subjected to single-marker analysis (Prasad et al., 2003).

## Results

### Phenotypic characteristics of the bean sprouts under different treatments

**Figure 1:**
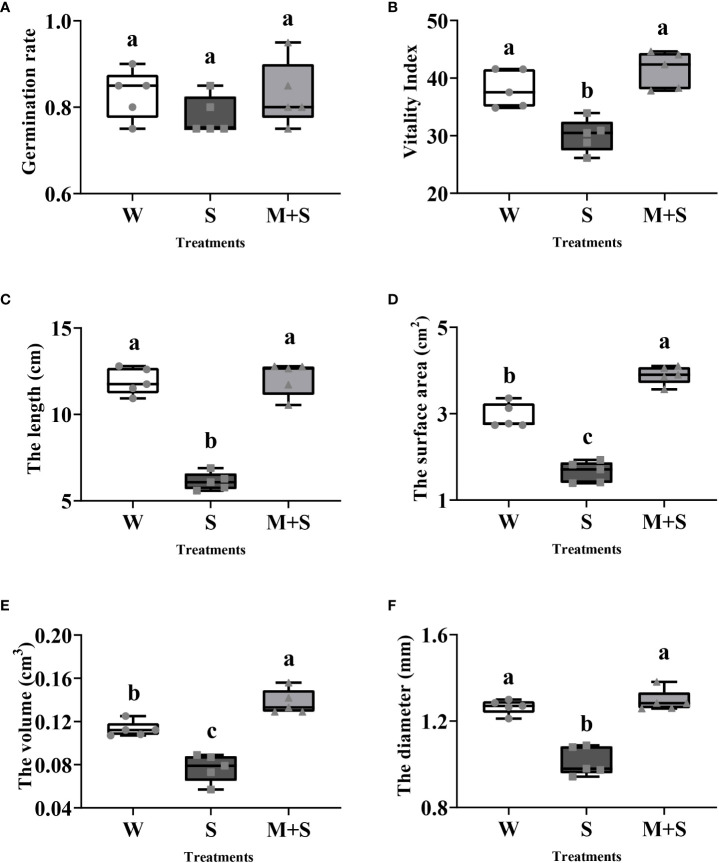
Phenotypic analysis of the common bean under W (water), S (salt), and M+S (melatonin with salt) treatments at the sprouting stage. Different lowercase letters in the same column indicate significant differences between the treatments (*P<0.05*). Each gray point represents the mean value of the technical replicates. **(A)** Germination rate; **(B)** Vitality index; **(C)** Length; **(D)** Surface area; **(E)** Volume; **(F)** Diameter.

The germination and phenotype indicators of the common bean were evaluated at the sprouting stage under three treatments (**Figure 1**). The germination results showed that although there was no significant difference in the germination rate among the three treatments, salt stress significantly decreased the vitality index, but this was significantly alleviated by exogenous melatonin (*P<0.05*). Salt stress inhibited the growth of common bean by significantly reducing (*P<0.05*) the length, surface area, volume, and diameter of common bean sprouts compared with CK (W). However, the exogenous melatonin alleviated the effects of salt stress on the common bean sprouts, as shown by the significant increase (*P<0.05*) in length, surface area, volume, and diameter of common bean sprouts under the M+S treatment. Collectively, these results illustrate that exogenous melatonin can enhance the phenotype of bean sprouts under salt stress.

The quality assessments of the RNA-Seq samples are shown in **Table S3**. The Q20 values of each sample were all greater than 94.93%, while the Q30 values were all greater than 88.21%. Moreover, the sequencing error rates of all samples were less than 0.02%, indicating that the samples were suitable for subsequent analysis. The raw data generated by RNA-Seq analysis have been uploaded to the NCBI (National Center for Biotechnology Information) database, with the accession number PRJNA603150.

**Figure 2:**
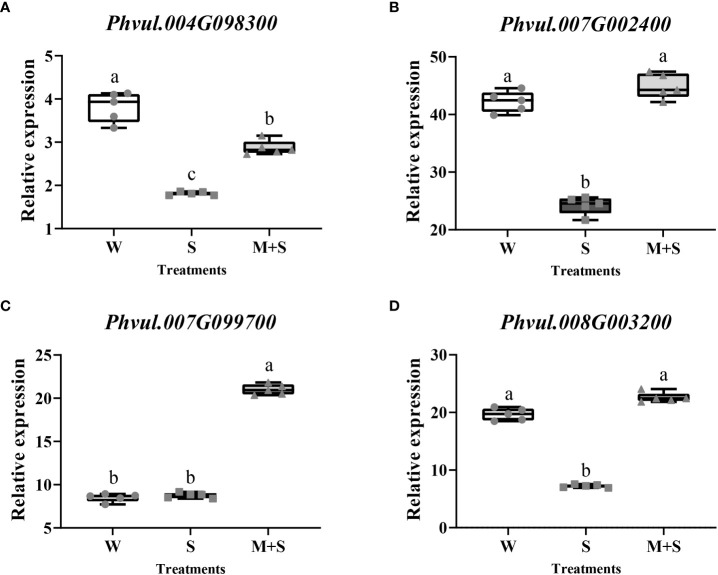
Gene expression analysis of the cell wall-related GO terms enriched in the common bean under W (water: CK), S (salt), and M+S (melatonin with salt) treatments at sprouting stage. Different lowercase letters in the same column indicate significant differences between the treatments (P<0.05). **(A)** Expression analysis of the *Phvul.004G098300*. **(B)** Expression analysis of the *Phvul.007G002400*. **(C)** Expression analysis of the *Phvul.007G099700*. **(D)** Expression analysis of the *Phvul.008G003200*.

A total of 217 DEGs were identified between S and M+S treatments based on log2FC≥1 and FDR<0.01, among which 150 genes were up-regulated (**Table S4**) while 67 were down-regulated (**Table S5**). We subjected these 217 DEGs to GO and KEGG enrichment analysis and found that five GO terms were significantly enriched (*Corrected P_Value<0.05*), among which three terms (GO:0009664, GO:0071669, and GO:0005199) were related to cell wall biosynthesis (**Table 1**). Therefore, four DEGs (*Phvul.004G098300*, *Phvul.007G002400*, *Phvul.007G099700*, and *Phvul.008G003200*) enriched in these cell wall-related GO terms were selected for qRT-PCR analysis, which the detailed information had been shown in **Table S6**. The results showed a significant change in the relative expression of these four DEGs, further illustrating the accuracy of the RNA-Seq data and the possible involvement of the cell wall-related terms in response to exogenous melatonin application (**Figure 2**).

**Table 1:** The GO enrichment analysis of DEGs.

GO_ID	Description	Term_type	Corrected P_Value
GO:0009664	Plant-type cell wall organization	Biological_process	0.0011
GO:0071669	Plant-type cell wall organization or biogenesis	Biological_process	0.0011
GO:0005199	Structural constituent of cell wall	Molecular_function	0.0050
GO:0030623	U5 snRNA binding	Molecular_function	0.0145
GO:0017069	SnRNA binding	Molecular_function	0.0156

**Figure 3:**
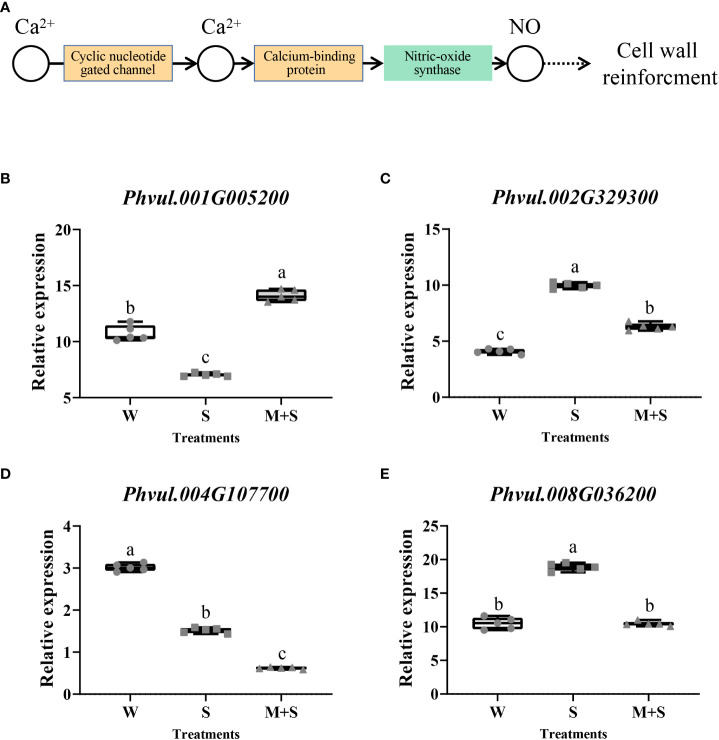
Analysis of the “Plant-pathogen interaction (pvu04626)” pathway and its related genes enriched in the KEGG pathway of the common bean under W (water: CK), S (salt), and M+S (melatonin with salt) treatments. Different lowercase letters in the same column indicate significant differences between the treatments (P<0.05). **(A)** “Plant-pathogen interaction’’ pathway; the brown part represents the enriched DEGs points. **(B)** Expression analysis of *Phvul.004G098300*. **(C)** Expression analysis of *Phvul.007G002400*. **(D)** Expression analysis of *Phvul.007G099700*. **(E)** Expression analysis of *Phvul.008G003200*.

In KEGG analysis, the “Plant-pathogen interaction (pvu04626)” pathway was the most enriched, with an enrichment P-value of 0.000. Cyclic nucleotide-gated channel and calcium-binding protein, which regulate nitric oxide (NO) for cell wall reinforcement, were the enriched points of this pathway (**Figure 3A**). Similarly, we selected four DEGs (*Phvul.001G005200*, *Phvul.002G329300*, *Phvul.004G107700*, and *Phvul.008G036200*) enriched in this pathway for qRT-PCR analysis, which the detailed information had been shown in **Table S7**. The results showed a significant change in the relative expression of these four genes, illustrating the accuracy of the RNA-Seq data. This suggested that the cell wall might be the response point to exogenous melatonin application (**Figures 3B–E**). The results of GO and KEGG analysis suggest that the cell wall may respond to melatonin and enhance salt stress in common bean.

### Regulation of melatonin and cell wall-related responses in the germplasm resources

**Figure 4:**
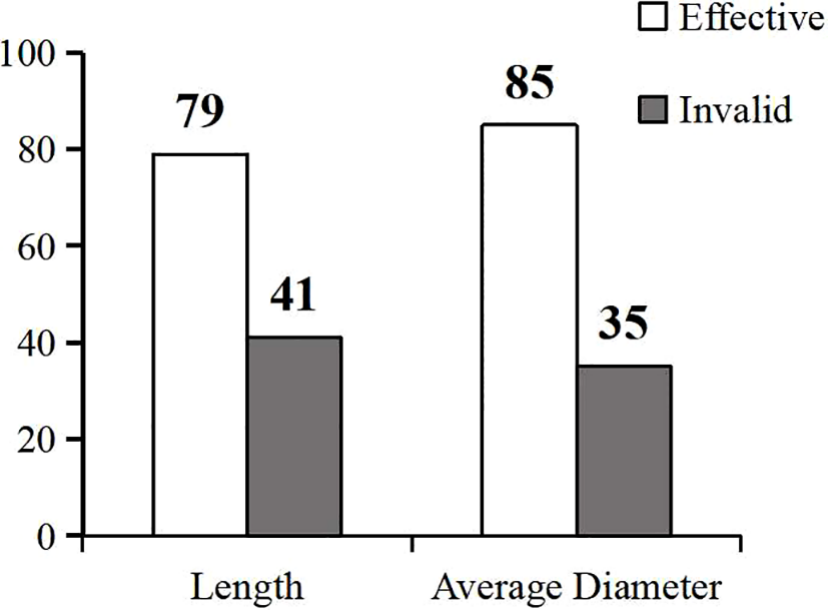
Validity analysis of exogenous melatonin in the common bean germplasm resources under salt stress. The gray columns represent the valid resources, while the white columns are the invalid resources.

To determine whether exogenous melatonin enhances the salt tolerance in common bean, we analyzed its change rate at the sprouting stage of the common bean under salt stress by measuring the length and diameter of the sprouts. The change rate of melatonin under salt stress ranged from -0.0739 to 2.5988, as calculated from the length of the sprouts (**Table S8**). We found that 79 germplasm resource materials were positively regulated (*P<0.05*), while five were negatively regulated by melatonin. The remaining 41 materials showed insignificant regulation by melatonin. The positive regulation rate of melatonin by the measured sprout length was 65.83%, while the change rate of melatonin under salt stress ranged from -0.3348 to 1.5485, as calculated from the diameter of the sprouts (**Table S9**). Furthermore, 85 materials were positively regulated (*P<0.05*). The positive regulation rate of melatonin by the measured sprout diameter was 70.83% (**Figure 4**). Altogether, these findings showed that the positive regulation rate of melatonin was more than 65%, suggesting that most varieties of common bean could have a positive response to melatonin.

To determine whether the cell wall is a regulatory pathway for melatonin processing, we selected 47 materials responding positively to the exogenous melatonin under salt stress for qRT-PCR analysis. S treatment served as the control. Four cell wall-related DEGs in GO terms and four DEGs enriched in the “Plant-pathogen interaction (pvu04626)” pathway by KEGG were selected for the analysis.

**Figure 5:**
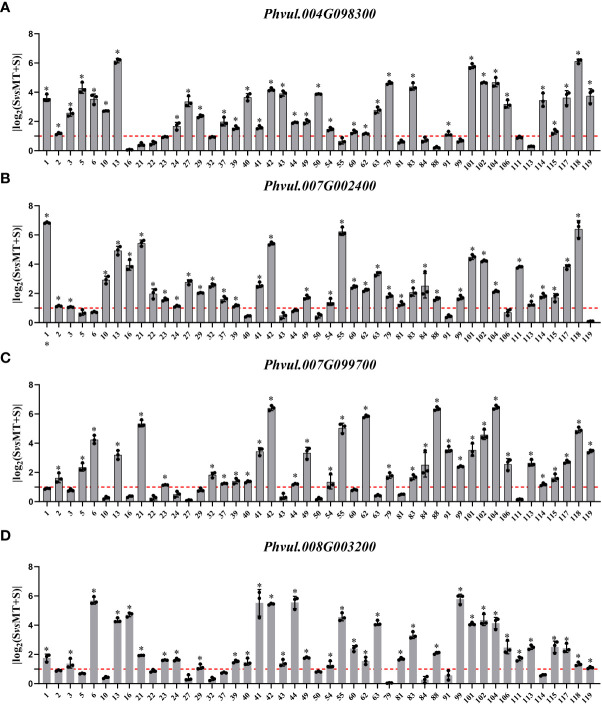
Effects of melatonin on the expression of genes associated with the cell wall-related GO terms in common bean germplasm resources under salt stress. The horizontal axis represents the number of resources, while the vertical axis shows the log_2_(fold change) values. Log_2_(fold change) value 1 (red line) is the critical value showing changes in the expression, while the columns higher than the red line represent the expression level with a significant change (*P<0.05*). * represents genes with significant different expression levels between S (salt) and M+S (melatonin and salt) treatments. **(A)** Expression analysis of *Phvul.004G098300*. **(B)** Expression analysis of *Phvul.007G002400*. **(C)** Expression analysis of *Phvul.007G099700*. **(D)** Expression analysis of *Phvul.008G003200*.

In the cell wall-related GO terms, the max log_2_FC value of *Phvul.004G098300* was 6.160 while the values of 35 materials were greater than one, with a positive response rate of 74.5%; The log_2_FC values of 38 materials were greater than one in the expression of *Phvul.007G002400*, with a positive response rate of 80.9%; Moreover, the expression of *Phvul.007G099700* showed that 33 materials had log_2_FC values greater than one and a positive response rate of 70.2%; while in *Phvul.008G003200*, the positive response rate with log_2_FC values greater than one was 74.5% (**Figure 5**).

**Figure 6:**
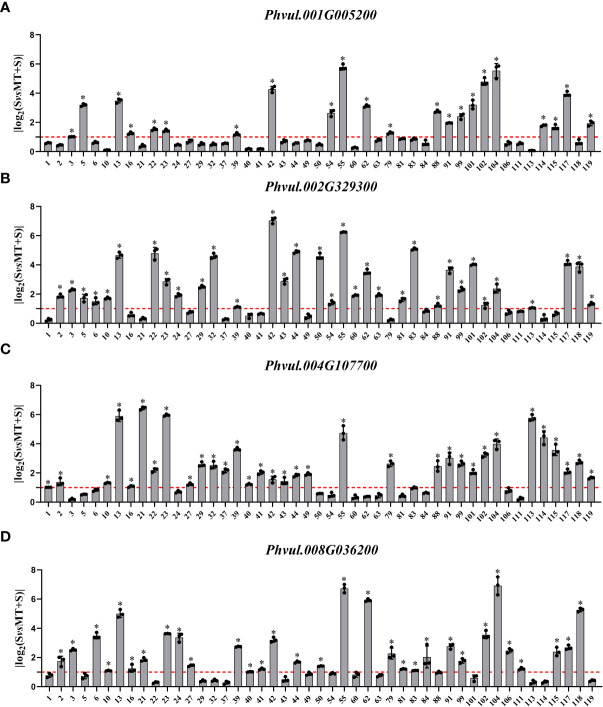
Effects of exogenous melatonin on the expression of genes associated with the “Plant-pathogen interaction (pvu04626)” pathway-related KEGG terms in common bean germplasm resources under salt stress. The horizontal axis represents the number of resources, while the vertical axis shows the log_2_(fold change) values. Log_2_(fold change) value 1 (red line) is the critical value showing changes in the expression, while the columns higher than the red line represent the expression level with a significant change (*P<0.05*). * represents genes with significant different expression levels between S (salt) and M+S (melatonin and salt) treatments. **(A)** Expression analysis of *Phvul.001G005200*. **(B)** Expression analysis of *Phvul.002G329300*. **(C)** Expression analysis of *Phvul.004G107700*. **(D)** Expression analysis of *Phvul.008G036200*.

In KEGG, the log_2_FC value of *Phvul.001G005200* ranged from 0.067 to 5.787: log_2_FC value of 22 materials was greater than one, and the positive response rate was 46.8%; log_2_FC value of 33 materials of *Phvul.002G329300* was greater than one with a positive response rate of 70.2%, the positive response rate of *Phvul.004G107700* was also 70.2%; log_2_FC value of 31 materials of *Phvul.008G036200* was greater than one, with the positive response rate of 66.0% (**Figure 6**). The expression data of the DEGs enriched in GO and KEGG showed that various DEGs had different positive response rates. More than half of the genes responded to melatonin. Altogether, these results indicate that the cell wall is an essential pathway in response to melatonin and enhances the salt tolerance of common bean at the sprouting stage.

### Construction and selection of markers

MISA locus was identified, and the markers were developed based on the common bean reference genome (PhaVulg1_0) from the ensemble database. A total of 135,015 molecular markers were developed, and linkage group 3 (LG3) contained the largest number of molecular markers (15,471). Additionally, eight LGs had more than 10,000 molecular markers (**Table S10**). The locations of DEGs in cell wall-related GO terms and the “Plant-pathogen interaction” pathway were selected as conditions using the 2000 bp interval adjacent to the DEGs. We screened 25 pairs of markers (**Table S11**) and found ten makers with polymorphisms that could be used for trait association analysis in response to exogenous melatonin. Furthermore, we selected 40 materials with or without positive melatonin regulation for association analysis and recorded the data as melatonin-responsive or non-melatonin-responsive phenotypes. The PCR-Acrylamide analysis of the ten makers was also recorded using different codes. The results showed that two markers: *Phvul.001G005200 (1)* and *Phvul.007G099700(2)*, had a significant association in response to melatonin (*P<0.05*), and could be used to predict whether common bean materials interact with exogenous melatonin under salt stress (**Table 2**).

**Table 2:** The molecular markers associated with exogenous melatonin response under salt stress.

The name of SSR makers	P-value	Significance
*Phvul.001G005200* (1)	0.048	*
*Phvul.001G067400* (1)	0.788	NS
*Phvul.002G329300* (2)	0.567	NS
*Phvul.003G110200* (2)	0.803	NS
*Phvul.004G107700* (1)	0.465	NS
*Phvul.005G026000* (2)	0.215	NS
*Phvul.007G002400* (2)	0.413	NS
*Phvul.007G084600* (2)	0.803	NS
*Phvul.007G099700* (2)	0.034	*
*Phvul.008G031800* (1)	0.917	NS

NS represents no significant difference between the two treatments, and * represents a significant difference between treatments (P<0.05).

## Discussion

Salt stress can cause excessive accumulation of ions, increase osmotic stress, stimulate overproduction of reactive oxygen species (ROS) and cause toxic effects in plants, thus inhibiting plant growth (Ubbens et al., 2018; Zhao et al., 2021). In this study, phenotypic indicators (such as length, surface area, volume, and diameter) revealed that salt stress inhibited the sprouting of common bean. Similarly, salt stress significantly inhibited primary radicle elongation (*P<0.05*) in maize (*Zea Mays*) at the sprouting stage (Li et al., 2021a). In common bean (*P. vulgaris*), the length, surface area, volume, and lateral root were reduced under salt stress compared with the control treatment (Zhang et al., 2021b). Salt stress also restricted the growth of Chinese Cabbage (*Brassica rapa*) by reducing the fresh weight and leaf area (Li et al., 2021b). Exogenous application of melatonin can be implemented to enhance plant tolerance to abiotic stress (Sun et al., 2021). Exogenous melatonin enhanced salt tolerance of *Arabidopsis thaliana* (Chen et al., 2017), orange (*Citrus aurantium*) (Kostopoulou et al., 2015), rice (*Oryza sativa*) (Li et al., 2017b), sweet potato (*Ipomoea batatas*) (Yu et al., 2018), tomato (*Solanum lycopersicum*) (Zhou et al., 2016), and watermelon (*Citrullus lanatus*) (Li et al., 2017a). In this study, phenotypic indicators of common bean seedlings were assessed after the seedlings were exposed to M+S treatment. The results showed that exogenous melatonin alleviated the growth inhibition caused by salt stress at the sprouting stage. This was consistent with previous studies on other plants (Zhan et al., 2019).

In this study, three GO terms related to the cell wall were identified by RNA-Seq, including plant-type cell wall tissue (GO:0009664), plant-type cell wall tissue, or related biomass synthesis (GO:0071669), and structural components of cell walls (GO:0005199) (**Table 1**). The “Plant-pathogen interaction’’ pathway (pvu04626) was the most enriched in KEGG analysis. The enriched DEGs suggested its role in the cell wall reinforcement (**Figure 3A**). Notably, qRT-PCR analysis of cell wall-related genes showed similar trends. Collectively, these results show that exogenous melatonin regulates salt stress by interacting with the genes related to the cell wall pathway. The cell wall has also been demonstrated to participate in salt stress (Endler et al., 2015). Under salt stress, the cell wall acts as the first barrier, which senses and initiates defense responses to increase plant salt tolerance (Zhao et al., 2021). Cell wall biosynthesis, especially remodeling under stress, is a common response to environmental changes (Endler et al., 2015). Several genes related to cell wall are regulated under stress (Zhao et al., 2020). The degree to which the cell wall responds to stress may affect the salt tolerance of plants (Zhang et al., 2021a). Various components of the cell wall may play a part in salt stress. For example, mutation in the genes involved in cellulose synthesis in the cell wall affects salt tolerance (Zhu et al., 2010). Also, proteins related to cellulose synthesis have been shown to accumulate in compartments in response to salt stress (Endler et al., 2015). The pectin content (cell wall composition) of two salt-extreme soya bean cultivars revealed the influence of cell wall on salt tolerance of roots (An et al., 2014). Lignin is thought to be closely related to salt stress. Some enzymes (such as phenylalaninammo-nialyase) in the lignin biosynthetic pathway were altered after salt treatment (Guo and Wang, 2009; Li et al., 2010). Increasing the strength of the cell wall could increase its ability to capture ions (Zhong and Ye, 2007) and reduce damage to the cell (Sun et al., 2020). The strengthening of the cell wall could increase the compactness of cellulose and change the physicochemical properties (such as pectin) of the cell wall components, which could intercept or adsorb more ions into the cell (Le Gall et al., 2015). Several studies have shown that exogenous melatonin can regulate cell wall tolerance to abiotic stress in plants. In cucumber, cell wall promoted copper ions (Cu^2+^) chelation, thus reducing their concentration in the cytoplasm and mitigating their harmful effects (Cao et al., 2019). Xie et al. (2020)reported that DEGs were significantly enriched in the GO terms of cell wall biogenesis (GO:0071554) in rice treated with exogenous melatonin under salt stress treatment. Additionally, exogenous melatonin enhanced stress tolerance in radish, and the cell wall-related terms, including cell wall macromolecule catabolic and cell wall macromolecule metabolic processes, were among the enriched pathways (Xu et al., 2020). Exogenous melatonin also increased antioxidant enzymes to regulate ROS and altered cell wall polysaccharides to mitigate Al^3+^ toxicity in wheat (Sun et al., 2020). In watermelon, exogenous melatonin regulated the expression of redox reaction and cell wall-related genes to enhance the tolerance of Cu^2+^ (Hu et al., 2020). Altogether, these studies indicate that exogenous melatonin regulates the cell wall to enhance plant tolerance to abiotic stress.

Although it has been reported that exogenous melatonin enhances abiotic stress tolerance in various crops, its effects vary in different crop varieties (Sun et al., 2020). Han et al. (2022)reported genotypic differences in the effect of exogenous melatonin on salt tolerance in common bean. Specifically, exogenous melatonin significantly enhanced the salt tolerance of some common bean varieties at the sprouting stage, except for the variety Xuliyabai. Similarly, not all common bean germplasm materials responded to melatonin treatment in the present study. Melatonin enhanced salt tolerance in some germplasm materials but did not affect some. The effective response rate to melatonin treatment was 65.83% by sprout length and 70.83% by sprout diameter, all above 65%. We found that most materials responded to exogenous melatonin, which enhanced their salt tolerance at the sprouting stage. Regulatory mechanisms by which exogenous melatonin confers salt tolerance in plants differ with plant species and genotypes (Sun et al., 2021). In this study, cell wall-related DEGs were found in “Naihua’’ common bean variety (GZ-YD014) through RNA-Seq, and 47 positive materials were used to evaluate the regulatory mechanisms associated with these DEGs. The results showed that the regulation rate of all cell wall-related DEGs was more than 46%, indicating that cell walls could interact with exogenous melatonin to enhance common bean salt tolerance at the sprouting stage.

Different varieties of plants exhibit different characteristics (such as plant height and tolerance to abiotic stress). Therefore, it is necessary to develop different types of identification and predictive analytics for practical applications, such as cell membrane stability (CMS) technique and prediction of molecular markers (Pih et al., 1997; Farooq and Azam, 2006). Molecular markers have co-dominance, high reproducibility, high polymorphism, low development cost, and rapidity (Biswas et al., 2020). These features make molecular markers ideal for genetic diversity surveys (Biswas et al., 2018), population structure analysis (Miyatake et al., 2019), genotyping (Chen et al., 2015), linkage mapping (Ambawat et al., 2016), and plant breeding studies. Molecular markers were utilized to identify the leaf- and seed-related traits in perilla (*Perilla frut escens*) (Lim et al., 2021). Also, Gllc 527 marker was used for marker-assisted selection of rust resistance in lentil (*Lens culinaris* Medikus) (Dikshit et al., 2016). However, there are a few reports on the markers associated with plant response to exogenous melatonin. In this study, two pairs of markers were found to be significantly associated with common bean response to exogenous melatonin. These two markers could be used as candidate markers to determine whether a particular common bean variety can respond to melatonin at the sprouting stage. This study provides useful data for screening of common bean varieties positively responding to exogenous melatonin.

## Conclusion

Exogenous melatonin increased the length, surface area, volume, and diameter of common bean sprouts under salt stress. GO, KEGG, RNA-Seq, and qRT-PCR analyses showed that the cell wall pathway was significantly enriched. Additionally, more than 65% of the germplasm materials were positively regulated by melatonin, as was shown by the length and diameter of common bean sprouts. Since the regulation rate of all cell wall-related DEGs was more than 46%, we suggest that cell walls might interact with exogenous melatonin to enhance the salt tolerance of the common bean at the sprouting stage. Furthermore, two pairs of makers were found to be associated with melatonin, which could be used as candidate markers for predicting how varieties of common bean respond to melatonin.

## Data availability statement

The datasets presented in this study can be found in online repositories. The names of the repository/repositories and accession number(s) can be found in the article/**Supplementary Material**.

## Author contributions

QZ: Data curation and writing original draft. BQ and G-dW: Data curation. W-jZ, XY and ML: Conceptualization and methodology. Z-gY and H-yS: Software; QZ and Y-lD: Formal data analysis and preparation of materials; J-dD, Y-lD and PJ: Conceptualization, data curation, revised the manuscript and funding acquisition. All authors contributed to the article and approved the submitted version.

## Acknowledgments

This study was financially supported by the National Key Research and Development Program (2020YFD1001402), the Research Project of Heilongjiang Bayi Agricultural University (XDB2011-02) and University Nursing Program for Young Scholars with Creative Talents in Heilongjiang Province (UNPYSCT-2018086).

## Conflict of interest

The authors declare that the research was conducted in the absence of any commercial or financial relationships that could be construed as a potential conflict of interest.

## Publisher’s note

All claims expressed in this article are solely those of the authors and do not necessarily represent those of their affiliated organizations, or those of the publisher, the editors and the reviewers. Any product that may be evaluated in this article, or claim that may be made by its manufacturer, is not guaranteed or endorsed by the publisher.
