# Supplementary material for: Exogenous melatonin enhances cell wall response to salt stress in common bean (Phaseolus vulgaris) and the development of the associated predictive molecular markers
Source: Front Plant Sci. 2022 Oct 17;13:1012186. doi: 10.3389/fpls.2022.1012186 (PMC9619082; doi:10.3389/fpls.2022.1012186)
Supplement: Supplementary file 1 [file Table_1.docx]

**Table S1: The detailed information on the 120 germplasm resources.**

| No. | Variety | No. | Variety | No. | Variety | No. | Variety |
| --- | --- | --- | --- | --- | --- | --- | --- |
| 1 | Pseudohongyun No.1 | 31 | Red face | 61 | Longyundou No.5 | 91 | Jianbian-purple safflower |
| 2 | White common bean(Japanese) | 32 | Small black Y98 | 62 | Milk circle | 92 | Team 4 local variety Y88 |
| 3 | Black Y94 | 33 | Shenmu chicken waist white | 63 | Purple flower | 93 | Longyun 10 |
| 4 | Yian red bean | 34 | Organic spotted bean | 64 | Columnar red flower | 94 | DZ37 |
| 5 | Milk pellet | 35 | Small red common bean | 65 | Longyun No.4 | 95 | Purple bean |
| 6 | British Red | 36 | Heihehua | 66 | Cloud white NN | 96 | Purple pink Phaseolus vulgaris |
| 7 | Organic spotted common bean | 37 | 15KF28 | 67 | adzuki common bean | 97 | Light Flower Common Bean |
| 8 | Milk flower bean | 38 | Big flower grain rice bean f1266 | 68 | Longjiang milk round | 98 | Y06×Y10 |
| 9 | Black Common | 39 | Red Kidney Beans | 69 | Red common bean Y4 | 99 | Non-pure color common bean |
| 10 | The New British Red | 40 | Yellow Oval Black Spot common bean | 70 | Red Island | 100 | Y22 |
| 11 | Black kidney bean Y81 | 41 | Common bean No.58×Y66 | 71 | Purple spotted common bean | 101 | Black common bean f2521 |
| 12 | Black common bean | 42 | Jilin black common bean Y3 | 72 | Syrian Flower | 102 | White cap bean |
| 13 | British Red common bean y84 | 43 | 15KF24 | 73 | Old flower point | 103 | Y2×Longyun 5 |
| 14 | Black common bean Y120 | 44 | Vyole84×F3378 | 74 | Milk flower common bean | 104 | Purple sand |
| 15 | 15ZY-033 | 45 | Dwarf oil bean | 75 | Brown long bean f3363 | 105 | Pure red bean |
| 16 | Black common bean Y92 | 46 | Bean F2002 | 76 | Safflower common bean f2525 | 106 | Lzl049 common bean |
| 17 | Black Kidney bean | 47 | Sun flower | 77 | Footprint | 107 | Japanese white |
| 18 | Y10×Y14 | 48 | 15ZY-193G | 78 | Old flower dot y138 | 108 | Small black common bean |
| 19 | Syriac Y91 | 49 | Dark red common F3362 | 79 | Unknown Kidney Bean | 109 | Long 29-1260 |
| 20 | White bean 3354 | 50 | Common bean f500 | 80 | Kenyun 2×Y29 | 110 | Y28 kenyun 1 |
| 21 | Black common bean 002 | 51 | Common beans with small yellow stripes | 81 | Y85 | 111 | Black bean |
| 22 | Black common bean Y135 | 52 | Purple Phaseolus vulgaris | 82 | White common face | 112 | Dragon 28-0650 |
| 23 | Farm variety Y96 | 53 | Heihe flower | 83 | Food common bean | 113 | American Red in Plant Protection Station |
| 24 | Shaksha F2517 | 54 | 15KF26 | 84 | Longjiang small common bean | 114 | Inner Mongolia safflower common bean |
| 25 | 15KF27 | 55 | ZX27 | 85 | Petal point | 115 | Red common bean |
| 26 | Phaseolus vulgaris | 56 | White hualian | 86 | Rice bean flower | 116 | Jiyun No.1 |
| 27 | S-135F3380 | 57 | White spotted face | 87 | Common bean | 117 | Lupus suspected vulgaris |
| 28 | Pseudo common bean | 58 | Black common bean f3394 | 88 | Red stubble common bean | 118 | GBX01 |
| 29 | Small common bean | 59 | England Red Y64 | 89 | Longjiang white common bean | 119 | GKUS01 |
| 30 | Dragon 28 | 60 | Dragon Red | 90 | Shandong jujube Y134 | 120 | Raw YD-300 |
