# Supplementary material for: Exogenous melatonin enhances cell wall response to salt stress in common bean (Phaseolus vulgaris) and the development of the associated predictive molecular markers
Source: Front Plant Sci. 2022 Oct 17;13:1012186. doi: 10.3389/fpls.2022.1012186 (PMC9619082; doi:10.3389/fpls.2022.1012186)
Supplement: Supplementary file 2 [file Table_2.docx]

**Table S2: The primers of qRT-PCR.**

| ID | Primer name | Primer sequence（5'to3'） |
| --- | --- | --- |
| 1 | *PvActin11*-qF | TGCATACGTTGGTGATGAGG |
| 2 | *PvActin11*-qR | AGCCTTGGGGTTAAGAGGAG |
| 3 | *Phvul.008G003200*F | GGTAACACTAAGAGCAATGGG |
| 4 | *Phvul.008G003200*R | GCCTTCAGAACAACCTCGTA |
| 5 | *Phvul.007G002400*F | AAGGTGCTACTGTTGAGGTTA |
| 6 | *Phvul.007G002400*R | ACTTTGTAGGTATGTTGAATG |
| 7 | *Phvul.007G099700*F | GCCATTATCCTTTTCAGCCTC |
| 8 | *Phvul.007G099700*R | ATGGGTAGTGGTAGGGAGGTG |
| 9 | *Phvul.002G329300*F | GACGAGATGAAGCGAGTT |
| 10 | *Phvul.002G329300*R | AATGAAGCCGTCGTGGTC |
| 11 | *Phvul.001G005200*F | GTCAAAAGGGAGGCGGAGT |
| 12 | *Phvul.001G005200*R | CCAAAGCGTCAGAAGCATT |
| 13 | *Phvul.008G036200*F | TCGGGTTTCGGACCGTATC |
| 14 | *Phvul.008G036200*R | GGTCAATCGCCAACGCC |
| 15 | *Phvul.004G107700*F | ATGATTGGGCAGTTCGGTG |
| 16 | *Phvul.004G107700*R | TTGTTCGCCGTCAGTGTCC |
| 17 | *Phvul.004G098300*F | TGCTTTTGCGTAGTGTCCC |
| 18 | *Phvul.004G098300*R | TTTGCTTCCGTTGTTTTCC |
