# Supplementary material for: Exogenous melatonin enhances cell wall response to salt stress in common bean (Phaseolus vulgaris) and the development of the associated predictive molecular markers
Source: Front Plant Sci. 2022 Oct 17;13:1012186. doi: 10.3389/fpls.2022.1012186 (PMC9619082; doi:10.3389/fpls.2022.1012186)
Supplement: Supplementary file 3 [file Table_3.docx]

**Table S3: The quality assessment of samples in RNA-Seq.**

| Sample  name | Raw reads | Clean reads | Error rate（%） | Q20（%） | Q30（%） |
| --- | --- | --- | --- | --- | --- |
| W_1_ | 55633866 | 53026610 | 0.01 | 96.84 | 92.16 |
| W_2_ | 50577884 | 48331946 | 0.02 | 96.69 | 91.84 |
| W_3_ | 52446224 | 50061634 | 0.02 | 96.68 | 91.86 |
| S_1_ | 41534300 | 40199494 | 0.02 | 94.93 | 88.25 |
| S_2_ | 50772136 | 49248286 | 0.02 | 95.1 | 88.47 |
| S_3_ | 48326882 | 46935654 | 0.02 | 95.13 | 88.53 |
| M+S_1_ | 51502046 | 49958064 | 0.02 | 95.08 | 88.43 |
| M+S_2_ | 51946416 | 50411968 | 0.02 | 94.93 | 88.21 |
| M+S_3_ | 45445652 | 44010506 | 0.02 | 95.3 | 88.83 |
