# Supplementary material for: Exogenous melatonin enhances cell wall response to salt stress in common bean (Phaseolus vulgaris) and the development of the associated predictive molecular markers
Source: Front Plant Sci. 2022 Oct 17;13:1012186. doi: 10.3389/fpls.2022.1012186 (PMC9619082; doi:10.3389/fpls.2022.1012186)
Supplement: Supplementary file 4 [file Table_4.docx]

| Table S4: The list of up-regulated genes in RNA-Seq analysis | |
| --- | --- |
| No. | Gene_ID |
| 1 | *Phvul.001G005200* |
| 2 | *Phvul.001G011500* |
| 3 | *Phvul.001G013200* |
| 4 | *Phvul.001G052100* |
| 5 | *Phvul.001G103800* |
| 6 | *Phvul.001G127400* |
| 7 | *Phvul.001G166300* |
| 8 | *Phvul.001G257600* |
| 9 | *Phvul.001G264400* |
| 10 | *Phvul.001G268700* |
| 11 | *Phvul.002G004800* |
| 12 | *Phvul.002G173900* |
| 13 | *Phvul.002G211800* |
| 14 | *Phvul.002G212100* |
| 15 | *Phvul.002G218300* |
| 16 | *Phvul.002G231800* |
| 17 | *Phvul.002G290700* |
| 18 | *Phvul.002G324800* |
| 19 | *Phvul.002G331900* |
| 20 | *Phvul.003G024400* |
| 21 | *Phvul.003G068600* |
| 22 | *Phvul.003G087300* |
| 23 | *Phvul.003G096700* |
| 24 | *Phvul.003G103900* |
| 25 | *Phvul.003G136200* |
| 26 | *Phvul.003G153200* |
| 27 | *Phvul.003G179800* |
| 28 | *Phvul.003G217500* |
| 29 | *Phvul.003G237800* |
| 30 | *Phvul.003G259900* |
| 31 | *Phvul.003G285800* |
| 32 | *Phvul.004G029100* |
| 33 | *Phvul.004G066300* |
| 34 | *Phvul.004G066500* |
| 35 | *Phvul.004G098300* |
| 36 | *Phvul.004G101800* |
| 37 | *Phvul.004G112900* |
| 38 | *Phvul.004G157300* |
| 39 | *Phvul.004G158000* |
| 40 | *Phvul.004G170600* |
| 41 | *Phvul.005G021100* |
| 42 | *Phvul.005G021200* |
| 43 | *Phvul.005G063900* |
| 44 | *Phvul.005G085400* |
| 45 | *Phvul.005G086200* |
| 46 | *Phvul.005G092900* |
| 47 | *Phvul.006G015200* |
| 48 | *Phvul.006G022200* |
| 49 | *Phvul.006G044800* |
| 50 | *Phvul.006G107300* |
| 51 | *Phvul.006G133600* |
| 52 | *Phvul.006G138600* |
| 53 | *Phvul.006G156000* |
| 54 | *Phvul.007G002400* |
| 55 | *Phvul.007G028700* |
| 56 | *Phvul.007G082800* |
| 57 | *Phvul.007G099700* |
| 58 | *Phvul.007G104800* |
| 59 | *Phvul.007G117500* |
| 60 | *Phvul.007G133600* |
| 61 | *Phvul.007G216700* |
| 62 | *Phvul.007G225100* |
| 63 | *Phvul.007G234300* |
| 64 | *Phvul.007G248400* |
| 65 | *Phvul.007G263900* |
| 66 | *Phvul.008G003200* |
| 67 | *Phvul.008G013100* |
| 68 | *Phvul.008G026600* |
| 69 | *Phvul.008G115600* |
| 70 | *Phvul.008G135400* |
| 71 | *Phvul.008G136400* |
| 72 | *Phvul.008G159000* |
| 73 | *Phvul.008G160400* |
| 74 | *Phvul.008G211800* |
| 75 | *Phvul.008G250600* |
| 76 | *Phvul.008G252000* |
| 77 | *Phvul.008G271700* |
| 78 | *Phvul.008G276200* |
| 79 | *Phvul.009G025300* |
| 80 | *Phvul.009G063700* |
| 81 | *Phvul.009G104900* |
| 82 | *Phvul.009G137600* |
| 83 | *Phvul.009G191800* |
| 84 | *Phvul.009G197000* |
| 85 | *Phvul.009G216100* |
| 86 | *Phvul.009G252700* |
| 87 | *Phvul.009G254100* |
| 88 | *Phvul.009G258900* |
| 89 | *Phvul.010G047000* |
| 90 | *Phvul.010G048000* |
| 91 | *Phvul.010G063700* |
| 92 | *Phvul.010G074700* |
| 93 | *Phvul.010G129600* |
| 94 | *Phvul.010G135700* |
| 95 | *Phvul.010G152300* |
| 96 | *Phvul.011G002000* |
| 97 | *Phvul.011G016300* |
| 98 | *Phvul.011G106300* |
| 99 | *Phvul.011G182900* |
| 100 | *Phvul.011G183500* |
| 101 | *Phvul.011G193800* |
| 102 | *Phvul.011G195700* |
| 103 | *Phvul.011G208200* |
| 104 | *Phvul.011G209700* |
| 105 | *Phvul.011G209800* |
| 106 | *Novel00013* |
| 107 | *Novel00071* |
| 108 | *Novel00124* |
| 109 | *Novel00185* |
| 110 | *Novel00186* |
| 111 | *Novel00188* |
| 112 | *Novel00194* |
| 113 | *Novel00214* |
| 114 | *Novel00219* |
| 115 | *Novel00220* |
| 116 | *Novel00233* |
| 117 | *Novel00251* |
| 118 | *Novel00264* |
| 119 | *Novel00270* |
| 120 | *Novel00272* |
| 121 | *Novel00273* |
| 122 | *Novel00298* |
| 123 | *Novel00303* |
| 124 | *Novel00320* |
| 125 | *Novel00323* |
| 126 | *Novel00333* |
| 127 | *Novel00366* |
| 128 | *Novel00395* |
| 129 | *Novel00421* |
| 130 | *Novel00435* |
| 131 | *Novel00436* |
| 132 | *Novel00450* |
| 133 | *Novel00452* |
| 134 | *Novel00460* |
| 135 | *Novel00484* |
| 136 | *Novel00491* |
| 137 | *Novel00495* |
| 138 | *Novel00496* |
| 139 | *Novel00498* |
| 140 | *Novel00525* |
| 141 | *Novel00531* |
| 142 | *Novel00542* |
| 143 | *Novel00575* |
| 144 | *Novel00616* |
| 145 | *Novel00621* |
| 146 | *Novel00685* |
| 147 | *Novel00690* |
| 148 | *Novel00705* |
| 149 | *Novel00807* |
| 150 | *Novel00840* |
