# Supplementary material for: Exogenous melatonin enhances cell wall response to salt stress in common bean (Phaseolus vulgaris) and the development of the associated predictive molecular markers
Source: Front Plant Sci. 2022 Oct 17;13:1012186. doi: 10.3389/fpls.2022.1012186 (PMC9619082; doi:10.3389/fpls.2022.1012186)
Supplement: Supplementary file 5 [file Table_5.docx]

| Table S5: The list of down-regulated genes in RNA-Seq analysis | |
| --- | --- |
| No. | Gene_ID |
| 1 | *Phvul.001G028100* |
| 2 | *Phvul.001G130200* |
| 3 | *Phvul.001G193200* |
| 4 | *Phvul.001G228500* |
| 5 | *Phvul.001G229200* |
| 6 | *Phvul.001G265300* |
| 7 | *Phvul.002G153900* |
| 8 | *Phvul.002G164500* |
| 9 | *Phvul.002G310200* |
| 10 | *Phvul.002G326000* |
| 11 | *Phvul.002G329300* |
| 12 | *Phvul.003G057300* |
| 13 | *Phvul.003G066700* |
| 14 | *Phvul.003G110200* |
| 15 | *Phvul.003G139200* |
| 16 | *Phvul.003G143400* |
| 17 | *Phvul.003G147500* |
| 18 | *Phvul.003G174800* |
| 19 | *Phvul.003G210000* |
| 20 | *Phvul.003G211400* |
| 21 | *Phvul.003G251000* |
| 22 | *Phvul.004G022700* |
| 23 | *Phvul.004G107700* |
| 24 | *Phvul.004G119600* |
| 25 | *Phvul.004G154900* |
| 26 | *Phvul.005G025500* |
| 27 | *Phvul.005G026000* |
| 28 | *Phvul.005G026100* |
| 29 | *Phvul.005G026800* |
| 30 | *Phvul.005G045900* |
| 31 | *Phvul.005G057400* |
| 32 | *Phvul.005G084800* |
| 33 | *Phvul.005G088600* |
| 34 | *Phvul.006G058600* |
| 35 | *Phvul.006G137800* |
| 36 | *Phvul.006G204100* |
| 37 | *Phvul.006G211100* |
| 38 | *Phvul.007G012700* |
| 39 | *Phvul.007G017100* |
| 40 | *Phvul.007G050200* |
| 41 | *Phvul.007G084600* |
| 42 | *Phvul.007G094300* |
| 43 | *Phvul.007G107400* |
| 44 | *Phvul.007G216200* |
| 45 | *Phvul.007G268900* |
| 46 | *Phvul.008G036200* |
| 47 | *Phvul.008G175700* |
| 48 | *Phvul.008G207300* |
| 49 | *Phvul.008G214200* |
| 50 | *Phvul.008G224600* |
| 51 | *Phvul.009G034000* |
| 52 | *Phvul.009G123300* |
| 53 | *Phvul.009G137000* |
| 54 | *Phvul.009G182200* |
| 55 | *Phvul.009G225000* |
| 56 | *Phvul.010G033300* |
| 57 | *Phvul.010G062900* |
| 58 | *Phvul.010G143000* |
| 59 | *Phvul.010G146300* |
| 60 | *Phvul.011G062400* |
| 61 | *Phvul.011G097400* |
| 62 | *Phvul.011G105600* |
| 63 | *Novel00111* |
| 64 | *Novel00530* |
| 65 | *Novel00559* |
| 66 | *Novel00646* |
| 67 | *Novel00781* |
