# Supplementary material for: Exogenous melatonin enhances cell wall response to salt stress in common bean (Phaseolus vulgaris) and the development of the associated predictive molecular markers
Source: Front Plant Sci. 2022 Oct 17;13:1012186. doi: 10.3389/fpls.2022.1012186 (PMC9619082; doi:10.3389/fpls.2022.1012186)
Supplement: Supplementary file 6 [file Table_6.docx]

Table S6: The detailed information of four different expressed genes (DEGs) in GO enrichment for qRT-PCR analysis

| No. | Gene_ID | PAC_Number | Description |
| --- | --- | --- | --- |
| 1 | Phvul.004G098300 | 37162478 | Extensin-like region |
| 2 | Phvul.007G002400 | 37165557 | Pollen proteins Ole e I like/Extensin-like region |
| 3 | Phvul.007G099700 | 37165157 | PRICHEXTENSN |
| 4 | Phvul.008G003200 | 37157757 | Pollen proteins Ole e I like |
