# Supplementary material for: Exogenous melatonin enhances cell wall response to salt stress in common bean (Phaseolus vulgaris) and the development of the associated predictive molecular markers
Source: Front Plant Sci. 2022 Oct 17;13:1012186. doi: 10.3389/fpls.2022.1012186 (PMC9619082; doi:10.3389/fpls.2022.1012186)
Supplement: Supplementary file 7 [file Table_7.docx]

Table S7: The detailed information of four different expressed genes (DEGs) in KEGG enrichment for qRT-PCR analysis

| No. | Gene_ID | PAC_Number | Description |
| --- | --- | --- | --- |
| 1 | Phvul.001G005200 | 37168298 | ENDOPLASMIN HOMOLOG |
| 2 | Phvul.002G329300 | 37176091 | EF-HAND CALCIUM-BINDING DOMAIN CONTAINING PROTEIN // SUBFAMILY NOT NAMED |
| 3 | Phvul.004G107700 | 37162233 | HEAT SHOCK PROTEIN 90 // SUBFAMILY NOT NAMED |
| 4 | Phvul.008G036200 | 37160007 | CYCLIC NUCLEOTIDE-GATED ION CHANNEL 2 |
