# Supplementary material for: Exogenous melatonin enhances cell wall response to salt stress in common bean (Phaseolus vulgaris) and the development of the associated predictive molecular markers
Source: Front Plant Sci. 2022 Oct 17;13:1012186. doi: 10.3389/fpls.2022.1012186 (PMC9619082; doi:10.3389/fpls.2022.1012186)
Supplement: Supplementary file 8 [file Table_8.docx]

**Table S8** The change rate of melatonin under salt stress calculated by sprout length.

| No. | Growth rate | No. | Growth rate | No. | Growth rate | No. | Growth rate |
| --- | --- | --- | --- | --- | --- | --- | --- |
| 1 | 0.2107* | 31 | 0.0813NS | 61 | 0.2358NS | 91 | 0.5508* |
| 2 | 0.1867* | 32 | 0.8569* | 62 | 0.3657* | 92 | 0.2299NS |
| 3 | 0.2558* | 33 | 0.0634NS | 63 | 0.4579* | 93 | 0.8888* |
| 4 | 0.1417NS | 34 | 0.6652* | 64 | 0.1424NS | 94 | 0.0095NS |
| 5 | 0.6039* | 35 | 0.1158NS | 65 | 0.2956* | 95 | 0.1196NS |
| 6 | 0.5066* | 36 | -0.0676NS | 66 | 0.2147* | 96 | 1.4507* |
| 7 | 0.2306* | 37 | 0.4832* | 67 | 0.0985NS | 97 | -0.0293NS |
| 8 | 0.4775* | 38 | 0.0999NS | 68 | 0.2361* | 98 | 0.4995* |
| 9 | 0.1932* | 39 | 0.3182* | 69 | 0.1276NS | 99 | 0.2368* |
| 10 | 0.1836* | 40 | 0.2793* | 70 | 0.2516* | 100 | 0.0568NS |
| 11 | 0.1959* | 41 | 0.4111* | 71 | 0.0168NS | 101 | 0.2746* |
| 12 | 0.5329* | 42 | 0.5190* | 72 | 0.2766* | 102 | 0.2889* |
| 13 | 0.6044* | 43 | 0.3592* | 73 | 0.1958* | 103 | 0.0601NS |
| 14 | 0.2142* | 44 | 0.3955* | 74 | 0.1138NS | 104 | 0.3319* |
| 15 | 0.2591* | 45 | 0.1225NS | 75 | 0.2316* | 105 | 0.1325NS |
| 16 | 0.9022* | 46 | 0.0265NS | 76 | 0.2134* | 106 | 0.7874* |
| 17 | 0.4954* | 47 | 0.0224NS | 77 | 0.1241NS | 107 | 0.2787* |
| 18 | 0.0908NS | 48 | 0.3378* | 78 | 0.0112NS | 108 | 0.0861NS |
| 19 | 0.2915* | 49 | 0.2420* | 79 | 0.3659* | 109 | -0.0245NS |
| 20 | 0.0417NS | 50 | 0.7449* | 80 | 0.1992* | 110 | 0.2271* |
| 21 | 0.3500* | 51 | 0.0323NS | 81 | 0.9879* | 111 | 0.2935* |
| 22 | 0.8547* | 52 | 0.0612NS | 82 | 0.0662NS | 112 | 0.6754* |
| 23 | 0.3436* | 53 | 0.2301* | 83 | 0.4965* | 113 | 0.4495* |
| 24 | 0.3109* | 54 | 0.4389* | 84 | 0.2042* | 114 | 1.1017* |
| 25 | 2.5988* | 55 | 0.2070* | 85 | 0.2077* | 115 | 0.7182* |
| 26 | 2.3146* | 56 | 0.2530* | 86 | 0.1091NS | 116 | 0.0597NS |
| 27 | 0.7542* | 57 | 0.1117NS | 87 | 0.3011* | 117 | 0.2321* |
| 28 | -0.0739NS | 58 | 0.1303NS | 88 | 0.3980* | 118 | 0.2344* |
| 29 | 0.3351* | 59 | 0.0873NS | 89 | 0.0820NS | 119 | 0.3516* |
| 30 | 0.0703NS | 60 | 0.1866* | 90 | 0.1385NS | 120 | 0.0722NS |

Note: NS represented no significant difference between two treatments, * represented a significant difference between treatments (*P<0.05*).
