# Supplementary material for: Exogenous melatonin enhances cell wall response to salt stress in common bean (Phaseolus vulgaris) and the development of the associated predictive molecular markers
Source: Front Plant Sci. 2022 Oct 17;13:1012186. doi: 10.3389/fpls.2022.1012186 (PMC9619082; doi:10.3389/fpls.2022.1012186)
Supplement: Supplementary file 9 [file Table_9.docx]

**Table S9** The change rate of melatonin under salt stress calculated by sprout diameter.

| No. | Growth rate | No. | Growth rate | No. | Growth rate | No. | Growth rate |
| --- | --- | --- | --- | --- | --- | --- | --- |
| 1 | 0.2118* | 31 | 0.0609NS | 61 | -0.1454NS | 91 | 0.3936* |
| 2 | 0.4304* | 32 | 0.2930* | 62 | 0.1934* | 92 | 0.2931* |
| 3 | 0.1944* | 33 | -0.0359NS | 63 | 0.1904* | 93 | 0.3724* |
| 4 | -0.1530NS | 34 | 0.5035* | 64 | 0.2621* | 94 | 0.0010NS |
| 5 | 0.1897* | 35 | 0.1023NS | 65 | 0.2670* | 95 | 0.0901NS |
| 6 | 0.2304* | 36 | 0.1690* | 66 | 0.4729* | 96 | 0.2133* |
| 7 | 0.1191NS | 37 | 0.3352* | 67 | 0.3015* | 97 | 0.0400NS |
| 8 | -0.3348* | 38 | -0.0432NS | 68 | 0.0741NS | 98 | 0.4032* |
| 9 | 0.1256NS | 39 | 0.1958* | 69 | 0.2171* | 99 | 0.1798* |
| 10 | 0.2142* | 40 | 0.2141* | 70 | 0.1915* | 100 | 0.0169NS |
| 11 | 0.2440* | 41 | 0.2656* | 71 | -0.1178NS | 101 | 0.1952* |
| 12 | 0.0857NS | 42 | 0.3197* | 72 | 0.1588* | 102 | 0.2735* |
| 13 | 0.2658* | 43 | 0.1497* | 73 | 0.1965* | 103 | 0.0375NS |
| 14 | 0.1870* | 44 | 0.3604* | 74 | 0.2905* | 104 | 0.2148* |
| 15 | 0.2757* | 45 | 0.1274NS | 75 | 0.2069* | 105 | 0.0691NS |
| 16 | 0.2660* | 46 | 0.0492NS | 76 | 0.1543* | 106 | 0.3666* |
| 17 | 0.2239* | 47 | -0.0316NS | 77 | 1.5485* | 107 | -0.0214NS |
| 18 | 0.1196NS | 48 | 0.0814NS | 78 | -0.1738NS | 108 | 0.0414NS |
| 19 | 0.3463* | 49 | 0.2935* | 79 | 0.3926* | 109 | -0.0177NS |
| 20 | 0.1741* | 50 | 0.2197* | 80 | 0.3675* | 110 | 0.2712* |
| 21 | 0.3260* | 51 | 0.0622NS | 81 | 0.6271* | 111 | 0.4738* |
| 22 | 0.3464* | 52 | -0.0062NS | 82 | 0.1889* | 112 | 0.2155* |
| 23 | 0.3890* | 53 | 0.2845* | 83 | 0.1984* | 113 | 0.1931* |
| 24 | 0.1818* | 54 | 0.2019* | 84 | 0.2491* | 114 | 0.1995* |
| 25 | 0.2347* | 55 | 0.2568* | 85 | 0.2683* | 115 | 0.1928* |
| 26 | 0.1952* | 56 | 0.2078* | 86 | -0.1498NS | 116 | 0.3009* |
| 27 | 0.3263* | 57 | 0.0758NS | 87 | 0.3792* | 117 | 0.4337* |
| 28 | 0.0963NS | 58 | 0.2137* | 88 | 0.1881* | 118 | 0.2379* |
| 29 | 0.2155* | 59 | 0.0845NS | 89 | 0.1079NS | 119 | 0.2243* |
| 30 | 0.1876* | 60 | 0.2474* | 90 | 0.1350NS | 120 | 0.1166NS |

Note: NS represented no significant difference between two treatm
