# Supplementary material for: Exogenous melatonin enhances cell wall response to salt stress in common bean (Phaseolus vulgaris) and the development of the associated predictive molecular markers
Source: Front Plant Sci. 2022 Oct 17;13:1012186. doi: 10.3389/fpls.2022.1012186 (PMC9619082; doi:10.3389/fpls.2022.1012186)
Supplement: Supplementary file 10 [file Table_10.docx]

**Table S10: Statistics on the number of SSR markers on each linkage group of common bean.**

| Linkage Group | Number of SSR marks |
| --- | --- |
| LG1 | 13481 |
| LG2 | 14680 |
| LG3 | 15471 |
| LG4 | 10736 |
| LG5 | 9634 |
| LG6 | 9279 |
| LG7 | 13608 |
| LG8 | 14529 |
| LG9 | 12917 |
| LG10 | 9623 |
| LG11 | 11057 |
