# Supplementary material for: Exogenous melatonin enhances cell wall response to salt stress in common bean (Phaseolus vulgaris) and the development of the associated predictive molecular markers
Source: Front Plant Sci. 2022 Oct 17;13:1012186. doi: 10.3389/fpls.2022.1012186 (PMC9619082; doi:10.3389/fpls.2022.1012186)
Supplement: Supplementary file 11 [file Table_11.docx]

**Table S11: The markers near cell wall differential genes in RNA-seq.**

| Gene_accession | No. | F or R | Sequence |
| --- | --- | --- | --- |
| *Phvul.001G005200* | 1 | F | TTTCTATGTATTTTTCTCCT |
|  | 1 | R | TTTTTCTTATTCTTTGTTCT |
|  | 2 | F | AAACAATACAACACCGCCAT |
|  | 2 | R | TTAGGTTCCTCTCTCTCACG |
| *Phvul.001G067400* | 1 | F | CTATAAACTTAGACAACGCT |
|  | 1 | R | TAATCCAAAATACCTATAAG |
| *Phvul.002G329300* | 1 | F | AAAACTAAAGGGTGAGGAAT |
|  | 1 | R | GAGGGTGTGATGATGTGG |
|  | 2 | F | AAGATGGGGTATGAAGAATG |
|  | 2 | R | CCAATCGCCAACCATAA |
|  | 3 | F | TTCCACTGGGATTCTATGTC |
|  | 3 | R | GGGTTTATTAACTTTCCTTTA |
| *Phvul.003G110200* | 1 | F | TAATCCTTAATGTGGACTAA |
|  | 1 | R | AAATAATCTTGAAATAAATC |
|  | 2 | F | AATGAAAATCCAGAAAAAAA |
|  | 2 | R | ATCTCAACGGAAAAGACGAA |
| *Phvul.004G098300* | 1 | F | AGTTTGACACGACTGCTATT |
|  | 1 | R | CGAAATACATTGATTCCCTT |
|  | 2 | F | TGGTAATAGTATGGAGGAGG |
|  | 2 | R | ATTGGCTGACAACTACATCT |
| *Phvul.004G107700* | 1 | F | GGTTGTCTATAAGTCACTAG |
|  | 1 | R | AATAATAAGTTTGTTTTTCT |
| *Phvul.005G026000* | 1 | F | TATCCCTTCTTATCAAAGTC |
|  | 1 | R | TATCCCTTCTTATCAAAGTC |
|  | 2 | F | TCTTTTTATCTTAAGTGTCG |
|  | 2 | R | TTTTCATTTTTTAGTTGTTT |
| *Phvul.007G002400* | 1 | F | TCATGCTCATACATACACAT |
|  | 1 | R | TTGTTCTTTAATTGAAAAAA |
|  | 2 | F | TCATTTCTTTTGTAGTACGT |
|  | 2 | R | ATTTGATCAGATTATTGGGG |
| *Phvul.007g084600* | 1 | F | CATGTAAGTCTCTCCTTATA |
|  | 1 | R | AAGTTGGATATTTTGGTATA |
|  | 2 | F | GAGGTGGTGGTGAAGGTGAT |
|  | 2 | R | TTTCTGTTAATGCTGATGCT |
| *Phvul.007G099700* | 1 | F | GACCTCGCTTTGCCACCA |
|  | 1 | R | TCTCCAGCACCACCCTTC |
|  | 2 | F | TACACTCAGGACAGGATT |
|  | 2 | R | CCTTCTTTCTTTCCTTCC |
|  | 3 | F | ACGCAACAATAAAAAAGA |
|  | 3 | R | TCCTCCAGTCCCTTCACC |
|  | 4 | F | TTTCAGCACGTTTTCTTA |
|  | 4 | R | TAGCATATCCTCGGTTAT |
| *Phvul.008G003200* | 1 | F | TCATGAGTACGTGCACAC |
|  | 1 | R | AGGAGGATATTAAAGGGT |
|  | 2 | F | AACAATGACACGTTTGAACG |
|  | 2 | R | GCTACGACTGGAAATACCCC |
| *Phvul.008G031800* | 1 | F | TTTTAGGTATAGAGAGGGAA |
|  | 1 | R | TTTTGAGCAAGAAAGTGTTA |
|  | 2 | F | TGACCCAATCTAAACTGAAG |
|  | 2 | R | AAGTAAACAATTATAACCAC |
